# Optimization of electrode material and geometry to enhance TTFields-like anticancer effects in MCF-7 breast cancer cells

**DOI:** 10.1038/s41598-026-65772-2

**Published:** 2026-08-13

**Authors:** Ghada M. Marie, Mamdouh M. Shawki, Seham Elabd, Nevan M. Fekry, Maisa E. Moustafa

**Affiliations:** 1 Medical Biophysics Department, Medical Research Institute, Alexandria University, Alexandria, Egypt; 2 Physiology Department, Medical Research Institute, Alexandria University, Alexandria, Egypt

**Keywords:** Breast cancer, Tumour treating fields, Silver electrodes, Gold electrodes, Stainless steel electrodes, Apoptosis, Oxidative stress, Cancer, Materials science, Nanoscience and technology, Oncology

## Abstract

Breast cancer is the most frequently diagnosed malignancy and a leading cause of cancer-related mortality among women worldwide. Tumor treating fields (TTFields) are a non-invasive anticancer modality based on low-intensity, intermediate-frequency alternating electric fields. In the present study, a conductive electrode-based TTFields-like in vitro exposure system was used to investigate how electrode material and geometry influence biological responses in breast cancer cells. Specifically, we examined the effects of electrode material (silver, gold, and stainless steel), surface area (0.5 and 1 cm^2^), and number of electrodes (two or four) on the response of human breast cancer cells (MCF-7) to TTFields-like exposure generated using conductive metallic electrodes (150 kHz, 2 V/cm). Cells were exposed once for 15 min using conductive plate electrodes in direct contact with the culture medium. Treatment outcomes were assessed using flow cytometric analysis of cell viability and apoptosis, quantitative PCR analysis of apoptotic regulators, measurement of total antioxidant capacity (TAC), and inductively coupled plasma mass spectrometry (ICP-MS) to quantify metal ion accumulation. Increasing electrode surface area produced a greater reduction in viable tumor cells than increasing electrode number across all materials. Silver electrodes induced the strongest cytotoxic and apoptotic effects, accompanied by a significant increase in the BAX/BCL-2 ratio, indicating activation of the mitochondrial apoptotic pathway. Notably, silver electrodes caused smaller reductions in TAC compared with gold and stainless-steel electrodes under equivalent conditions, despite detectable metal ion accumulation. These findings demonstrate that electrode material and surface area are critical determinants of cellular response to TTFields-like exposure, highlighting a balance between anticancer efficacy and oxidative stress in vitro.

**Supplementary Information:**

The online version contains supplementary material available at 10.1038/s41598-026-65772-2.

## Introduction

Breast cancer is the most frequently diagnosed malignancy and remains a leading cause of cancer-related mortality among women worldwide, accounting for a substantial proportion of global cancer incidence and deaths^1^. Despite considerable advances in early diagnosis and therapeutic strategies, breast cancer continues to present major clinical challenges due to its biological heterogeneity, complex etiology, and the frequent emergence of treatment resistance^2^. Conventional treatment modalities, including surgery, radiotherapy, and chemotherapy, remain the cornerstone of clinical management; however, their invasive nature and associated adverse effects often compromise patients’ quality of life^3^. These limitations have driven increasing interest in alternative and non-invasive therapeutic approaches.

Exposure of biological cells to electric fields can induce structural changes in the cell membrane, leading to increased permeability through the formation of transient or permanent pores, a phenomenon known as electropermeabilization^4^. This principle has been widely applied in electrochemotherapy, where electric fields enhance the intracellular uptake of chemotherapeutic agents, improving treatment efficacy^5,6^. Another electric-field-based approach, irreversible electroporation, employs higher electric-field doses that produce permanent membrane disruption and subsequent cell death without the use of drugs or radiation^7,8^. In contrast to electrochemotherapy and irreversible electroporation, which rely on membrane permeabilization induced by short-duration, high-intensity electric pulses, TTFields employ low-intensity, intermediate-frequency alternating electric fields that primarily target intracellular processes associated with cancer cell division^9–11^.

Tumor treating fields (TTFields) represent a distinct and clinically established electric field–based therapy that employs low-intensity (1–3 V/cm), intermediate-frequency (100–300 kHz) alternating electric fields to inhibit tumor growth^9^. TTFields are applied through externally positioned electrodes and exert their anticancer effects through multiple mechanisms, including disruption of mitotic spindle formation, interference with microtubule dynamics, impairment of DNA repair, induction of autophagy and apoptosis, modulation of membrane permeability, and inhibition of cancer cell migration and invasion^10,11^. Importantly, TTFields demonstrate selectivity toward cancer cells due to differences in cell size, shape, and dielectric properties compared with normal cells, allowing effective tumor suppression while sparing surrounding healthy tissues^12–14^. The biological efficacy of TTFields is influenced not only by field intensity and frequency but also by electric-field distribution and uniformity, which determine the extent of interaction between alternating electric fields and intracellular charged or polarizable structures^15^.

In breast cancer models, previous studies have demonstrated that human breast carcinoma cells are particularly sensitive to TTFields at frequencies around 150 kHz and field intensities of approximately 1.75–3 V/cm^16^. However, effective treatment often requires prolonged exposure durations, suggesting that optimal treatment parameters for TTFields have not yet been fully defined. Furthermore, when TTFields are applied in combination with other therapeutic modalities, it remains difficult to delineate the precise contribution of electric field exposure to disease control^16^.

In the present study, the term TTFields-like exposure is used to distinguish the experimental system from clinically approved TTFields technology. Clinical TTFields are delivered through insulated transducer arrays designed to generate alternating electric fields while minimizing direct electrode–electrolyte interactions. In contrast, the current in vitro system employed conductive metallic electrodes positioned above the cell culture. Consequently, although the generated electric field shared key physical characteristics with TTFields, including intermediate-frequency alternating current exposure (150 kHz), additional electrochemical effects such as metal ion release may occur. Therefore, the present system is described as TTFields-like rather than a clinical TTFields platform.

While substantial research has focused on the biological mechanisms and optimization of clinical TTFields systems^17–19^, comparatively little attention has been given to conductive electrode-based TTFields-like exposure platforms. Unlike clinical TTFields, where insulated transducer arrays minimize direct electrode–tissue interactions, conductive electrode systems may introduce additional variables related to electrode material, geometry, ion release, and electrochemical activity. Understanding how these factors influence biological responses is essential for interpreting experimental TTFields-like studies and for optimizing electric-field-based anticancer approaches.

While substantial research has focused on optimizing TTFields frequency, intensity, and treatment duration, considerably less attention has been given to electrode-related parameters, including electrode material, surface area, and configuration. These factors are expected to influence electric field distribution, dose density, and biological response. In experimental systems employing conductive electrodes in direct contact with biological media, additional electrochemical phenomena, such as metal ion release and oxidative stress, may further modulate cellular outcomes. However, the relative contribution of these electrode-dependent effects to anticancer efficacy and cellular stress responses remains poorly understood.

To our knowledge, no previous study has systematically evaluated the combined effects of electrode material, surface area, and electrode number on the biological response of breast cancer cells to TTFields-like exposure while simultaneously quantifying oxidative stress and metal ion accumulation. Accordingly, the present study aimed to identify optimal electrode-related conditions for generating TTFields-like exposure using conductive electrodes, to achieve equal or enhanced anticancer effects over shorter exposure times while limiting oxidative stress. Using the human breast cancer cell line MCF-7, we systematically investigated the influence of electrode material (silver, gold, and stainless steel), electrode surface area, and electrode number on cell viability and apoptosis, apoptosis regulators, oxidative stress, and metal ion accumulation, thereby providing insight into electrode optimization strategies for electric field–based cancer therapies. MCF-7 cells were selected because they represent one of the most extensively characterized human breast adenocarcinoma cell lines models and have been widely used in studies investigating electric-field-based anticancer therapies, allowing direct comparison with previously published TTFields-related studies.

## Materials and methods

### Cell line and culture conditions

The human breast adenocarcinoma cell line MCF-7 was used as an in vitro model of estrogen receptor–positive breast cancer from the National Institute of Oncology, Cairo, Egypt. Cells were cultured on collagen-coated tissue culture dishes (Sarstedt AG & Co., Nümbrecht, Germany) in a complete medium of high glucose Dulbecco’s Modified Eagle’s Medium (DMEM) with L-Glutamine (Lonza, Brussels, Belgium), supplemented with 10% fetal bovine serum (FBS) (Sigma, Berlin, Germany), 1% penicillin and streptomycin (Biochrom GmbH). Cells were kept under standard cell culture conditions at 37 °C in a humidified CO_2_ incubator containing 5% CO_2_. Cells were subcultured at approximately 70–80% confluence using trypsin–EDTA and were used for experiments between defined passage numbers^20–25^ to ensure cellular consistency.

### Electric field exposure system

**Fig. 1:**
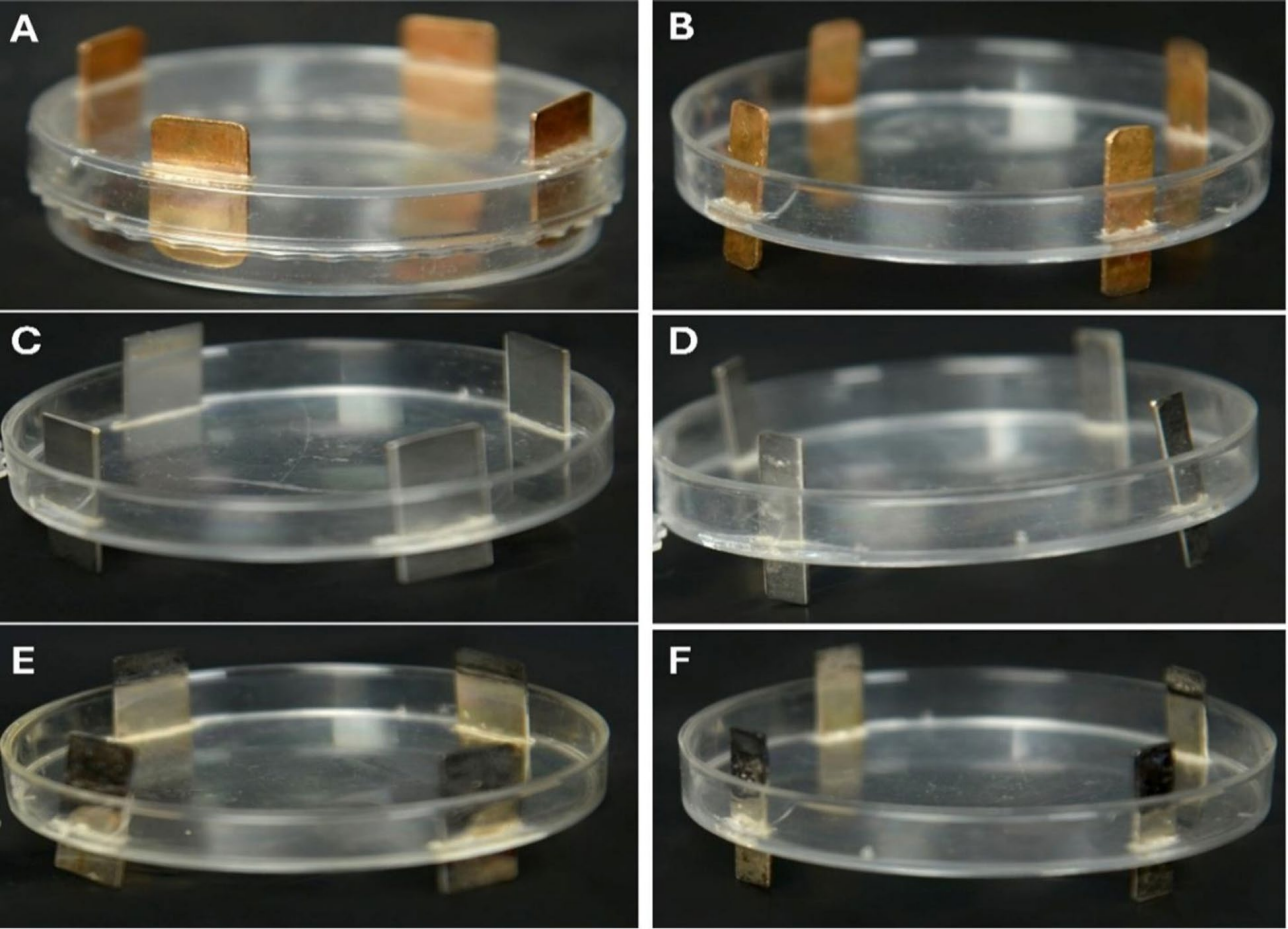
The different electrode materials and surface areas. **A, B**: Gold electrodes of 1 cm^2^ and 0.5 cm^2^ surface area, respectively. **C, D**: Silver/ silver chloride electrodes of 1 cm^2^ and 0.5 cm^2^ surface area, respectively. **E, F**: Stainless steel electrodes of 1 cm^2^ and 0.5 cm^2^ surface area, respectively.

TTFields-like exposure was created using a custom-designed system consisting of parallel conductive plate electrodes connected to an alternating current signal generator (CALTEK, CA1640P-02 function generator/counter, serial number: 06mg0676, USA). The setup was configured to deliver intermediate-frequency sinusoidal electric pulses at a frequency of 150 kHz and a field intensity of 2 V/cm, parameters previously shown to be effective in breast cancer cell models^20^. Two holes were drilled in the lid of the cell culture dish; through them, the electrodes were inserted and fixed. Each electrode connects to one output terminal of the function generator. To evaluate electrode-dependent effects, electrodes made from silver, gold, or stainless steel were used (Fig. 1). Two surface areas (0.5 cm^2^ and 1 cm^2^) and two configurations (two or four electrodes) were tested. The distance between electrodes was kept consistent across all conditions at 5 cm to ensure a similar field distribution. The spacing was determined by the dimensions of the Petri dish and the arrangement of the electrodes mounted on the dish cover, while allowing consistent electrode placement in all experimental groups. Keeping the inter-electrode distance constant ensured that electrode material, electrode number, and electrode surface area are the primary variables under investigation. Signal verification was performed using an oscilloscope connected to the electrode terminals during operation of the exposure system. The recorded waveform demonstrated stable sinusoidal alternating-current generation at 150 kHz, with symmetrical positive and negative half-cycles, stable amplitude, and no observable DC offset or waveform distortion. The applied voltage was maintained at approximately 10 V and monitored using a voltmeter. Based on the fixed inter-electrode distance of 5 cm, the corresponding electric field intensity was estimated to be approximately 2 V/cm. The electrodes were positioned in direct contact with the culture medium, enabling electric field propagation across the cell monolayer.

Silver, gold, and stainless-steel electrodes were selected because they represent conductive materials with distinct electrical and electrochemical characteristics that are widely used in biomedical and bioelectronic applications. Silver possesses very high electrical conductivity and is commonly used in biomedical electrodes and bio-signal acquisition systems. Gold is considered a standard bioelectrode material because of its excellent electrical conductivity, high corrosion resistance, chemical stability, and proven biocompatibility. Stainless steel is extensively used in implantable and medical devices owing to its mechanical strength, corrosion resistance, and acceptable electrical conductivity. The substantial differences among these materials in conductivity, electrochemical stability, and potential ion release characteristics make them suitable candidates for investigating how electrode material influences TTFields-like biological responses. Because silver exhibits the highest electrical conductivity among the tested materials, followed by gold and stainless steel, we hypothesized that electrode material may influence current distribution, electric-field delivery, and electrochemical interactions at the electrode–medium interface, thereby affecting cellular responses^21–23^.

### Experimental design

The experimental design employed a metal-only control strategy to isolate the biological effects of electric field exposure from those arising from electrode–medium contact and potential electrochemical interactions. For each electrode material and configuration, cells were exposed to conductive plate electrodes placed in direct contact with the culture medium for 15 min either in the presence or absence of the electric field. The metal-only condition, in which electrodes were applied without electric field exposure, served as the reference control for all analyses. Accordingly, all biological outcomes were expressed relative to the corresponding metal-only control of identical electrode material, surface area, and number.

TTFields-like exposure was applied once at a frequency of 150 kHz and a field intensity of 2 V/cm, for 15 min. This exposure duration was selected to assess whether electrode optimization could enhance anticancer effects over short exposure periods while minimizing prolonged electric stress. All exposures were performed at room temperature, The temperature of the culture dishes was recorded immediately before and after each exposure session using an infrared thermometer (RH-9601, Ronix Company, Made in China), and no appreciable temperature increase was observed. The exposure parameters (150 kHz, 2 V/cm, 15 min) were selected based on the established biophysical principles of TTFields and published studies demonstrating biological activity of intermediate-frequency alternating electric fields in breast cancer models. Specifically, 150 kHz falls within the reported optimal frequency range for breast cancer cells, while electric field intensities of approximately 1–3 V/cm have been shown to exert antiproliferative and pro-apoptotic effects without inducing significant thermal damage. A 15-min exposure period was chosen to provide measurable biological responses while minimizing heating, excessive electrochemical reactions at the electrode interface, and extensive loss of cell viability that could compromise downstream molecular and biochemical analyses^11,24,25^.

Electrode-dependent effects were systematically evaluated by varying electrode material (gold, silver/silver chloride, and stainless steel), electrode surface area (0.5–1 cm^2^), and electrode number (two or four). For each material, cells were exposed using two- or four-electrode configurations with surface areas of 0.5 cm^2^ and 1 cm^2^, resulting in a total of twelve electric field–exposed experimental conditions, each paired with its corresponding control. Following TTFields-like exposure, cells were returned to standard culture conditions (37 °C, 5% CO₂) and incubated for 48 h prior to flow cytometry, total antioxidant capacity (TAC), gene expression, and ICP-MS analyses.

This design enabled a controlled assessment of how electrode material and geometry modulate cellular responses to TTFields-like exposure while accounting for electrode-associated effects.

### Flow cytometry-apoptosis analysis

Following electric field exposure, cells were harvested and processed for flow cytometric analysis. Cell viability and apoptosis were assessed using annexin V and propidium Iodide solution staining according to the manufacturer’s protocol. Briefly, 1 × 10^6^ cells were washed with phosphate-buffered saline (PBS), resuspended in binding buffer, and incubated with annexin V–FITC and PI in the dark at room temperature. Samples were analyzed using a flow cytometer (FACScan flow cytometer, Becton Dickinson, San Diego, Calif, USA). Data were acquired and recorded using the Cellquest software, and data were processed to quantify viable, early apoptotic, late apoptotic, and necrotic cell populations^26^.

### Quantitative real-time PCR analysis of apoptotic and cell cycle–related genes

Quantitative real-time polymerase chain reaction (qRT-PCR) was performed to evaluate the molecular effects of TTFields-like exposure on apoptotic and cell cycle regulatory pathways. Total RNA was extracted from treated and corresponding metal-only control cells using a commercial RNA isolation kit (RNeasy mini kit (cat. nos. 74104 and 74106, Qiagen)) according to the manufacturer’s instructions. RNA concentration and purity were assessed spectrophotometrically by measuring absorbance at 260 and 280 nm, and only samples with acceptable purity ratios were used for subsequent analysis.

Complementary DNA (cDNA) was synthesized from equal amounts of total RNA using a reverse transcription kit (Enzynomics Co., Ltd., Korea, category number: RT220) following the supplier’s protocol. Quantitative PCR amplification was carried out using gene-specific primers for apoptotic markers (BAX and BCL-2) and cell cycle–related genes (cyclin A, cyclin B and cyclin E), which were selected to assess electric field–induced modulation of mitochondrial apoptosis and cell cycle progression, respectively. Primer sequences for all tested genes, including the housekeeping gene (GAPDH), are listed in Table 1.

Quantitative real-time PCR was performed by (Refurbished Applied Biosystems 7500 Fast Real Time PCR system) under optimized cycling conditions consisting of initial denaturation followed by repeated cycles of denaturation, annealing, and extension. Relative gene expression levels were normalized to the housekeeping gene and calculated using the comparative Ct (2⁻ΔΔCt) method^27,28^. The BAX/BCL-2 expression ratio was used as an indicator of mitochondrial apoptotic pathway activation, while changes in cyclin gene expression were used to evaluate electric field–induced alterations in cell cycle regulation.

**Table 1:** Primer sequence of tested genes.

Gene name	Primer sequence
GAPDH	F: 5′-GTCAGTGGTGGACCTGACCT-3′
	R: 5′-CACCACCCTGTTGCTGTAGC-3′
BAX	F: ′-TTCTGACGGCAACTTCAACTG-3′
	R: 5′-TGAGGAGTCTCACCCAACCA-3′
BCL-2	F: 5′-GACGCTTTGCCACGGTGGTG-3′
	R: 5′-GGGGCAGGCATGTTGACTTCAC-3′
Cyclin A	F: 5′-GTCACCACATACTATGGACATG-3′
	R: 5′-AAGTTTTCCTCTCAGCACTGAC-3′
Cyclin B	F: 5′-CGAAACTTAACACAGAAAATAAGGCC-3′
	R: 5′-CTTTCACATATTCGCTACAGAGG-3′
Cyclin E	F: 5′-GTCCTGGCTGAATGTATACATGC-3′
	R: 5′-CCCTATTTTGTTCAGACAACATGGC-3′

### Measurement of total antioxidant capacity (TAC)

Following exposure to TTFields-like exposure for the indicated duration, cells were incubated under standard culture conditions and subsequently processed for oxidative stress analysis. Cell monolayers were washed with PBS and lysed to obtain protein extracts. The total protein concentration in each sample was determined using the Bradford assay according to the method described by Bradford^29^, with bovine serum albumin (BSA) used as the calibration standard.

TAC was then measured in the obtained protein extracts using a colorimetric assay based on a single electron transfer (SET) mechanism, which quantifies the cumulative antioxidant activity of the sample. TAC values were normalized to the total protein content of each sample and expressed relative to the corresponding control group for each electrode material and configuration.

### Inductively coupled plasma mass spectrometry (ICP-MS)

The accumulation of elemental gold, silver, and iron in MCF-7 cells was quantified using inductively coupled plasma mass spectrometry (ICP-MS). Following treatment, culture plates were rinsed twice with PBS to remove extracellular ions. Cells were detached using trypsin, collected by centrifugation at 400 × g for 5 min, and the supernatant was discarded. Cell pellets were resuspended in 400 µL of culture medium, and viable cells were counted using the trypan blue exclusion assay. Samples were adjusted to a final volume of 2 mL and stored at − 20 °C until analysis.

Before ICP-MS measurement, samples were thawed and subjected to acid digestion to ensure complete mineralization of cellular material. For digestion, 144 µL of 69% nitric acid (trace metal grade), 810 µL of 37% hydrochloric acid (trace metal grade), and 20 µL of internal standard were added to each sample, vortexed, and incubated for 30 min at room temperature. The volume of each digested sample was then adjusted to 10 mL with ultrapure deionized water, vortexed, and transferred to an autosampler for ICP-MS analysis.

### Statistical analysis

All experiments were performed using three independent biological replicates. For each experimental condition, a single exposed cell culture was subsequently used for flow cytometry, TAC, gene expression analysis, and ICP-MS measurements. The entire experimental procedure was independently repeated three times. For qRT-PCR analysis, each biological replicate was analyzed in triplicate technical replicates. Data were expressed as mean ± standard deviation (SD). Statistical analyses were performed using IBM SPSS Statistics version 22.0 (IBM Corp., Armonk, NY, USA). One-way analysis of variance (ANOVA) followed by Tukey’s honestly significant difference (HSD) post hoc test was used for flow cytometry, TAC, and ICP-MS datasets. Gene expression data were analyzed using the Kruskal-Wallis test. A p-value < 0.05 was considered statistically significant.

## Results

Preliminary analysis demonstrated that metal-only control groups corresponding to gold, silver/silver chloride, and stainless steel electrodes produced statistically indistinguishable results across all assessed parameters (cell viability, apoptosis, gene expression, TAC, and metal accumulation). Accordingly, metal-only control data were pooled and are collectively referred to as the control group in the Results section.

###  Effects of TTFields-like exposure on cell viability and apoptosis

**Fig. 2:**
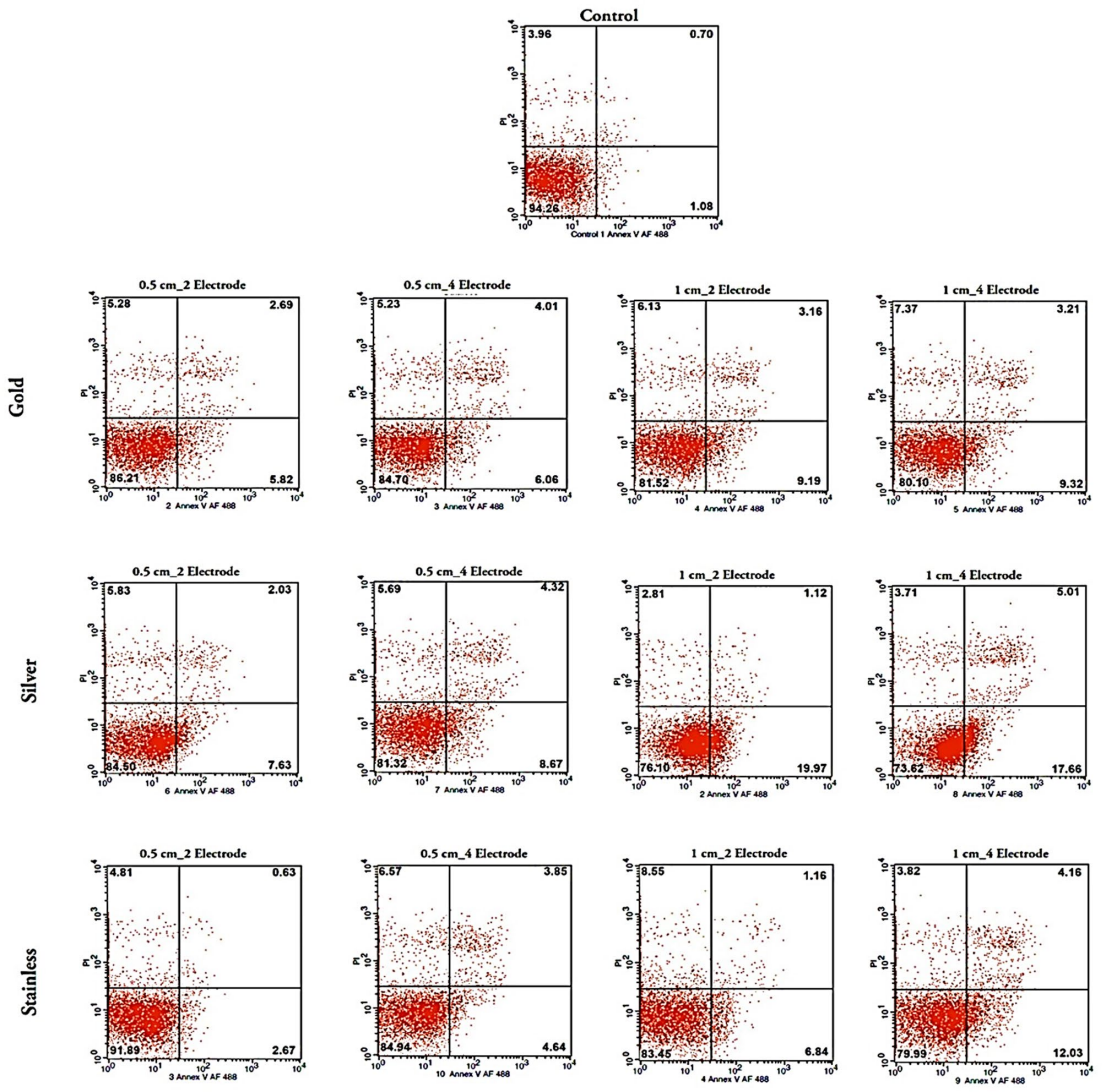
Representative flow cytometry analysis of MCF-7 cell fate following TTFields-like exposure using different electrode materials and configurations. Representative Annexin V-FITC/propidium iodide (PI) dot plots are shown for control cells and all experimental groups. Quadrants represent viable cells (Annexin V⁻/PI⁻), early apoptotic cells (Annexin V⁺/PI⁻), necrotic cells (Annexin V⁻/PI⁺), and late apoptotic/dead cells (Annexin V⁺/PI⁺).

**Fig. 3:**
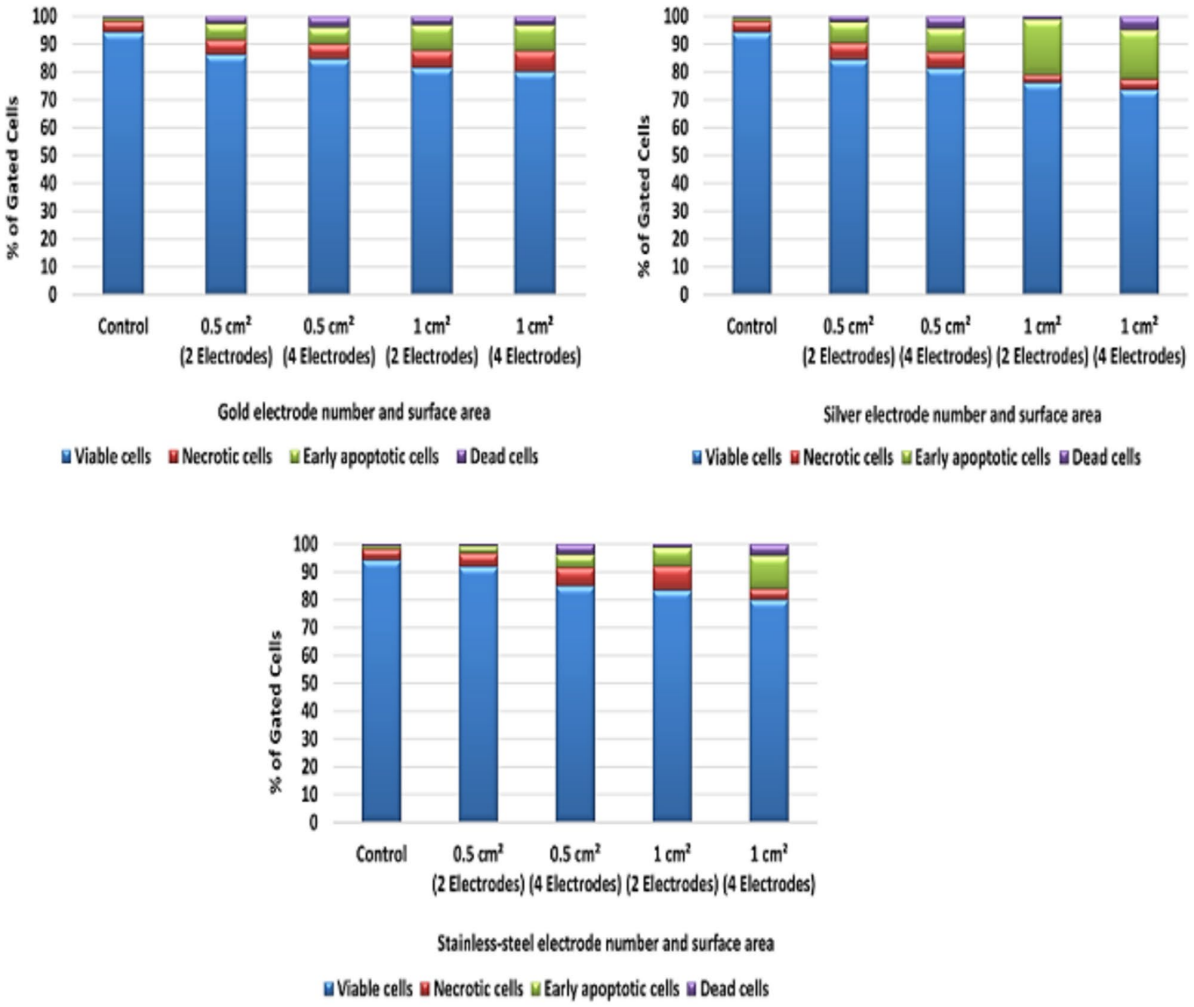
Viability distribution of MCF-7 cells following TTFields-like exposure conducted by gold, silver, and stainless-steel electrodes. The data represent the percentage of gated cells. All exposed groups differed significantly from the control (*p* < 0.001).

Flow cytometric analysis was performed to quantify changes in the percentage of viable MCF-7 cells after exposure to TTFields-like applied through gold, silver, and stainless-steel electrodes. Representative flow cytometry dot plots for the control and all TTFields-like exposure conditions are presented in Fig. 2. Quantitative analysis of the gated cell populations, including viable, necrotic, early apoptotic, and dead cells, is summarized in Table 2 and illustrated in Fig. 3.

Across all electrode materials and configurations, exposure to TTFields-like resulted in a highly significant reduction in viable (normal) cells, accompanied by significant increases in apoptotic, necrotic, and dead cell populations, compared with the control group (*p* < 0.001 for most conditions).

####  Viable (normal) cell population

The control group exhibited a high percentage of viable cells (94.26 ± 0.02%). Following electric field exposure, viable cell percentages declined significantly in all experimental groups.

At identical electrode numbers and surface areas, silver electrodes consistently produced the greatest reduction in viable cells, followed by gold and stainless-steel electrodes. For example, at 1 cm^2^ surface area with four electrodes, viable cell percentages decreased to (73.62 ± 0.04%) for silver, compared with (80.10 ± 0.50%) for gold and (79.99 ± 0.01%) for stainless steel, corresponding to reductions of (20.64%), (14.16%), and (14.27%), respectively (*p* < 0.001).

Within each electrode material, increasing electrode surface area from 0.5 to 1 cm^2^ resulted in a larger reduction in viable cells than increasing the number of electrodes from two to four. This trend was consistent across gold, silver, and stainless-steel electrodes.

#### Apoptotic cell population

The apoptotic cell fraction in control samples was minimal (1.08 ± 0.04%). Electric field exposure induced a marked and statistically significant increase in apoptotic cells in all exposed groups.

Silver electrodes produced the strongest apoptotic response under all configurations. At 1 cm^2^ surface area with four electrodes, apoptotic cell percentages reached (19.97 ± 0.36%), significantly higher than those observed with gold (12.03 ± 0.33%) and stainless-steel electrodes (9.32 ± 0.05%) (*p* < 0.001). The increase in apoptotic cells was evident even at lower surface areas and electrode numbers, but intensified with increasing surface area.

Gold electrodes induced a moderate apoptotic response, whereas stainless-steel electrodes consistently produced the lowest apoptotic percentages across all conditions.

#### Necrotic cell population

Necrotic cell percentages in the control group were low (3.96 ± 0.01%). Following electric field exposure, necrosis increased in most electrode configurations, although its magnitude and pattern differed from those of apoptosis.

No uniform pattern was found between the three electrodes’ materials. For example, at 1 cm^2^ with two electrodes, necrotic cell percentages reached (8.55 ± 0.03%) for stainless steel, compared with (6.13 ± 0.09%) for gold and (2.81 ± 0.04%) for silver (*p* < 0.001). While at 1 cm^2^ with four electrodes, necrotic cell percentages reached (3.82 ± 0.05%) for stainless steel, compared with (7.37 ± 0.08%) for gold and (3.71 ± 0.01%) for silver (*p* < 0.001).

#### Dead cell population

The percentage of dead cells in the control group was minimal (0.70 ± 0.02%). Electric field exposure significantly increased dead cell percentages across all electrode materials and configurations.

Silver electrodes again exhibited the most pronounced effect at higher surface areas and electrode numbers, reaching (5.01 ± 0.04%) dead cells at 1 cm^2^ with four electrodes, compared with (3.21 ± 0.04%) for gold and (4.16 ± 0.03%) for stainless steel (*p* < 0.001).

#### Comparative electrode performance

Direct comparison of electrode materials at identical electrode numbers and surface areas revealed a consistent hierarchy of effectiveness, with silver electrodes producing the strongest overall cytotoxic effect, followed by gold, and then stainless steel. This ranking was evident across reductions in viable cells and increases in apoptotic and dead cell populations.

**Table 2:** Flow cytometric analysis of MCF-7 cell fate following TTFields-like exposure using different electrode materials and configurations.

Group	Viable (%)	Apoptotic (%)	Necrotic (%)	Dead (%)
**Control**	94.26 ± 0.02	1.08 ± 0.04	3.96 ± 0.01	0.70 ± 0.02
**Gold electrodes**				
0.5 cm^2^ – 2 electrodes	86.21^bc^ ± 0.20	5.82^bc^ ± 0.12	5.28 ± 0.44	2.69^bc^ ± 0.05
0.5 cm^2^ – 4 electrodes	84.70^b^ ± 0.32	6.06^bc^ ± 0.29	5.23^bc^ ± 0.08	4.01^bc^ ± 0.04
1 cm^2^ – 2 electrodes	81.52^bc^ ± 0.03	9.19^bc^ ± 0.52	6.13^bc^ ± 0.09	3.16^bc^ ± 0.01
1 cm^2^ – 4 electrodes	80.10^b^ ± 0.50	12.03^bc^ ± 0.33	7.37^bc^ ± 0.08	3.21^bc^ ± 0.04
**Silver electrodes**				
0.5 cm^2^ – 2 electrodes	84.50^ac^ ± 0.01	7.63^ac^ ± 0.01	5.83 ± 0.29	2.03^ac^ ± 0.01
0.5 cm^2^ – 4 electrodes	81.32^ac^ ± 0.03	8.67^ac^ ± 0.03	5.69^ac^ ± 0.03	4.32^ac^ ± 0.02
1 cm^2^ – 2 electrodes	76.10^ac^ ± 0.02	17.66^ac^ ± 0.13	2.81^ac^ ± 0.04	1.12^a^ ± 0.05
1 cm^2^ – 4 electrodes	73.62^ac^ ± 0.04	19.97^ac^ ± 0.36	3.71^a^ ± 0.01	5.01^ac^ ± 0.04
**Stainless-steel electrodes**				
0.5 cm^2^ – 2 electrodes	91.89^ab^ ± 0.03	2.67^ab^ ± 0.01	4.81 ± 0.03	0.63^ab^ ± 0.03
0.5 cm^2^ – 4 electrodes	84.94^b^ ± 0.02	4.64^ab^ ± 0.05	6.57^ab^ ± 0.01	3.85^ab^ ± 0.04
1 cm^2^ – 2 electrodes	83.45^ab^ ± 0.05	6.84^ab^ ± 0.03	8.55^ab^ ± 0.03	1.16^a^ ± 0.01
1 cm^2^ – 4 electrodes	79.99^b^ ± 0.01	9.32^ab^ ± 0.05	3.82^a^ ± 0.05	4.16^ab^ ± 0.03

Data are presented as mean ± SD (*n* = 3).

Superscript letters indicate statistically significant pairwise differences between electrode materials under identical electrode surface area and electrode number.

a: *p* ≤ 0.05 versus gold electrodes.

b: *p* ≤ 0.05 versus silver electrodes.

c: *p* ≤ 0.05 versus stainless-steel electrodes.

Absence of a superscript letter indicates no statistically significant difference between the compared groups (*p* > 0.05).

### Effect of TTFields-like exposure on the expression of apoptotic and cell cycle–related genes

To elucidate the molecular mechanisms underlying TTFields-like cytotoxicity, the expression of key apoptotic markers (BAX and BCL-2) and cell cycle regulatory genes (cyclins A, B, and E) was evaluated in MCF-7 cells following exposure using different electrode materials, surface areas, and electrode numbers.

#### Effect on cell cycle–related genes (cyclins A, B, and E)

The expression levels of cyclin A, cyclin B, and cyclin E were quantified by qRT-PCR following electric field exposure under all experimental conditions. As summarized in Table 3, no statistically significant differences were detected in the expression of any of the tested cyclins between exposed groups and their corresponding controls (*p* > 0.05). Moreover, no significant variation was observed among different electrode materials (gold, silver, or stainless steel), electrode numbers, or surface areas.

These findings indicate that TTFields-like exposure under the present experimental conditions did not induce detectable alterations in cell cycle progression at the transcriptional level. Accordingly, the observed reduction in cell viability cannot be attributed to cyclin-mediated cell cycle arrest, suggesting that alternative mechanisms are primarily responsible for the anticancer effects.

**Table 3:** Relative mRNA expression of cyclin A, cyclin B, and cyclin E in MCF-7 cells following TTFields-like exposure using different electrode materials and configurations.

Group	Cyclin A	Cyclin B	Cyclin E
**Control**	1.01 ± 0.10	1.01 ± 0.10	1.01 ± 0.10
**Gold electrodes**			
0.5 cm^2^ – 2 electrodes	0.90 ± 0.08	1.04 ± 0.11	0.96 ± 0.07
0.5 cm^2^ – 4 electrodes	1.14 ± 0.04	0.99 ± 0.14	0.89 ± 0.10
1 cm^2^ – 2 electrodes	0.89 ± 0.12	0.88 ± 0.05	0.91 ± 0.06
1 cm^2^ – 4 electrodes	1.03 ± 0.14	1.12 ± 0.11	0.92 ± 0.09
**Silver electrodes**			
0.5 cm^2^ – 2 electrodes	0.90 ± 0.07	1.06 ± 0.14	1.00 ± 0.08
0.5 cm^2^ – 4 electrodes	1.07 ± 0.09	1.09 ± 0.06	1.07 ± 0.07
1 cm^2^ – 2 electrodes	0.97 ± 0.08	1.04 ± 0.14	1.05 ± 0.14
1 cm^2^ – 4 electrodes	1.08 ± 0.09	0.89 ± 0.08	1.01 ± 0.11
**Stainless-steel electrodes**			
0.5 cm^2^ – 2 electrodes	1.11 ± 0.14	1.00 ± 0.08	1.02 ± 0.12
0.5 cm^2^ – 4 electrodes	0.98 ± 0.05	0.95 ± 0.07	1.05 ± 0.09
1 cm^2^ – 2 electrodes	1.04 ± 0.09	0.96 ± 0.04	0.97 ± 0.13
1 cm^2^ – 4 electrodes	1.14 ± 0.09	1.08 ± 0.12	0.93 ± 0.07

Data are expressed as mean ± SD (*n* = 3). No statistically significant differences were detected between exposed groups and their corresponding metal-only controls or among different electrode materials and configurations (ANOVA, *p* > 0.05).

#### Effect of TTFields-like exposure on apoptotic gene expression

**Fig. 4:**
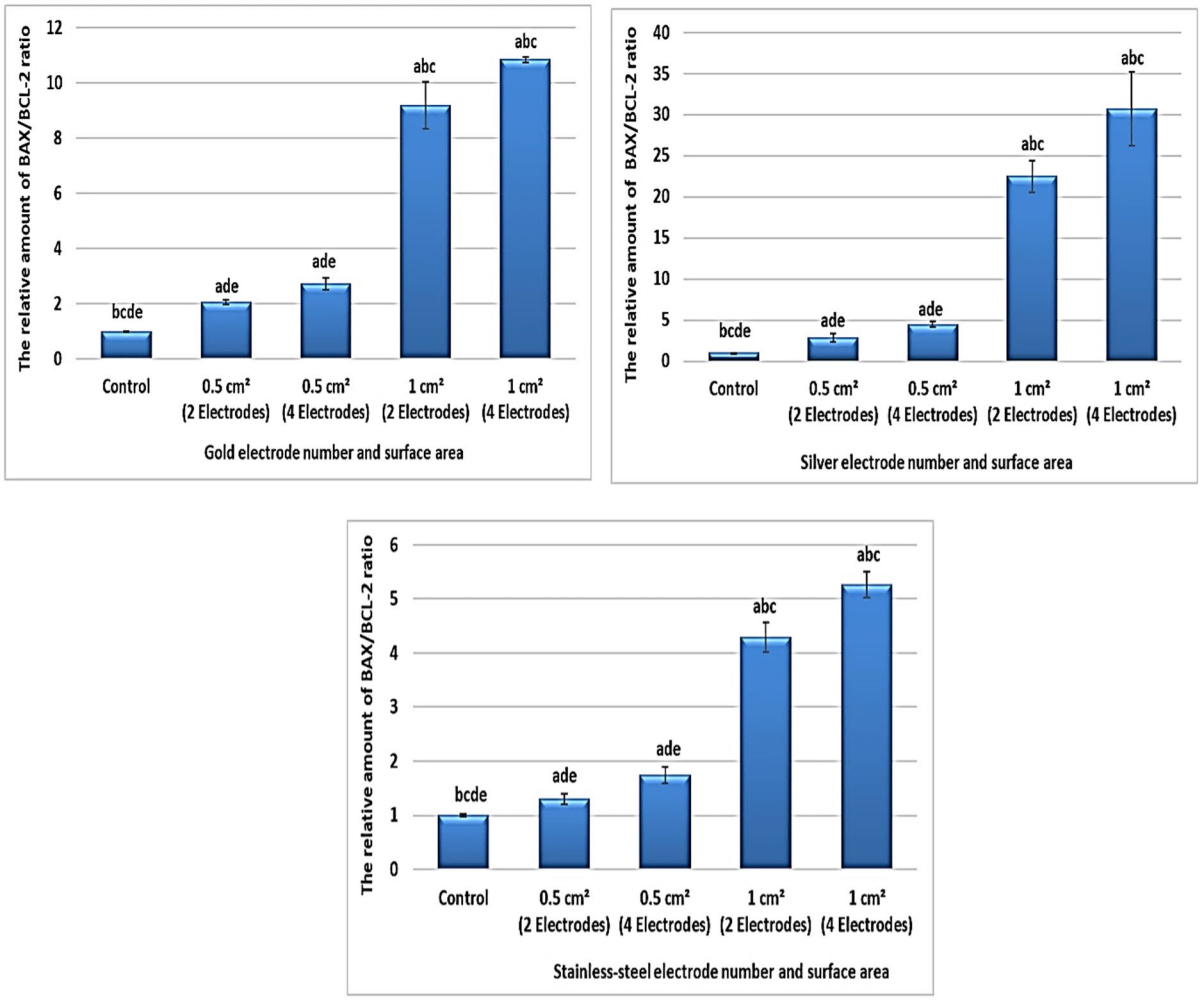
BAX/BCL-2 ratio of MCF-7 cells following TTFields-like exposure conducted by gold, silver, and stainless-steel electrodes. The significance between groups is determined by the Kruskal-Wallis test; data were expressed in mean ± standard error. a indicates *p* ≤ 0.05 compared to the **control** group. b indicates *p* ≤ 0.05 compared to the  **0.5 cm**^**2**^ **/ 2 electrodes** group. c indicates *p* ≤ 0.05 compared to the ** 0.5 cm**^**2**^ **/ 4 electrodes** group. d indicated *p* ≤ 0.05 compared to the  **1 cm**^**2**^ **/ 2 electrodes** group. e indicates *p* < 0.05 compared to the **1 cm**^**2**^ **/ 4 electrodes** group.

Quantitative real-time PCR analysis showed a significant, electrode-dependent alteration of apoptotic gene expression in MCF-7 cells after exposure to TTFields-like. Across all electrode materials and setups, exposure caused a notable increase in the pro-apoptotic gene BAX and a simultaneous decrease in the anti-apoptotic gene BCL-2 compared with the control group (*p* ≤ 0.05). Apoptotic signalling was further evaluated using the BAX/BCL-2 expression ratio (Fig. 4). Silver electrodes produced the highest pro-apoptotic response, with the BAX/BCL-2 ratio reaching (30.68 ± 4.46) at 1 cm^2^ with four electrodes, significantly exceeding the corresponding values observed for gold and stainless-steel electrodes (*p* ≤ 0.05). Detailed BAX, BCL-2, and the BAX/BCL-2 ratio for gold, silver, and stainless-steel electrode expression values are provided in Supplementary Tables S1–S3, respectively.

Exposure using gold electrodes produced a progressive increase in apoptotic signaling with increasing electrode surface area and number. BAX expression increased from (1.74 ± 0.10) at 0.5 cm^2^ with two electrodes to (4.31 ± 0.13) at 1 cm^2^ with four electrodes, while BCL-2 expression decreased from (0.85 ± 0.05) to (0.40 ± 0.01) under the same conditions (Table S1). Consequently, the BAX/BCL-2 ratio increased significantly from (2.05 ± 0.08) to (10.86 ± 0.10), with larger electrode surface area exerting a more pronounced effect than electrode number.

Silver electrodes induced the strongest apoptotic response among all tested materials. As shown in Table S2, BAX expression increased significantly from 1.98 ± 0.10 at 0.5 cm^2^ with two electrodes to (7.52 ± 0.26) at 1 cm^2^ with four electrodes, while BCL-2 expression decreased sharply from (0.70 ± 0.09) to (0.24 ± 0.06). This resulted in a dramatic elevation of the BAX/BCL-2 ratio, reaching 30.68 ± 4.46 under the largest surface area and highest electrode number condition, significantly exceeding the corresponding values observed for gold and stainless-steel electrodes (*p* ≤ 0.05).

In contrast, stainless-steel electrodes elicited the weakest apoptotic response. Although BAX expression increased and BCL-2 expression decreased significantly compared with control (Table S3), the magnitude of change was substantially lower than that observed with gold and silver electrodes. The highest BAX/BCL-2 ratio achieved using stainless-steel electrodes was (5.27 ± 0.24) at 1 cm^2^ with four electrodes, indicating a comparatively modest activation of apoptotic signaling.

Across identical electrode geometries, the magnitude of apoptotic gene modulation followed a consistent hierarchy in which silver is higher than gold, and gold is higher than stainless steel. Increasing electrode surface area from 0.5 cm^2^ to 1 cm^2^ produced a significantly greater effect on BAX upregulation and BCL-2 downregulation than increasing electrode number from two to four, regardless of electrode material (*p* ≤ 0.05). These findings confirm that electrode material and surface area are dominant determinants of TTFields-like apoptotic signaling in MCF-7 cells. This pattern also mirrors the flow cytometry findings and confirms that apoptosis is a major contributor to electric field–induced cytotoxicity.

### Total antioxidant capacity

**Fig. 5:**
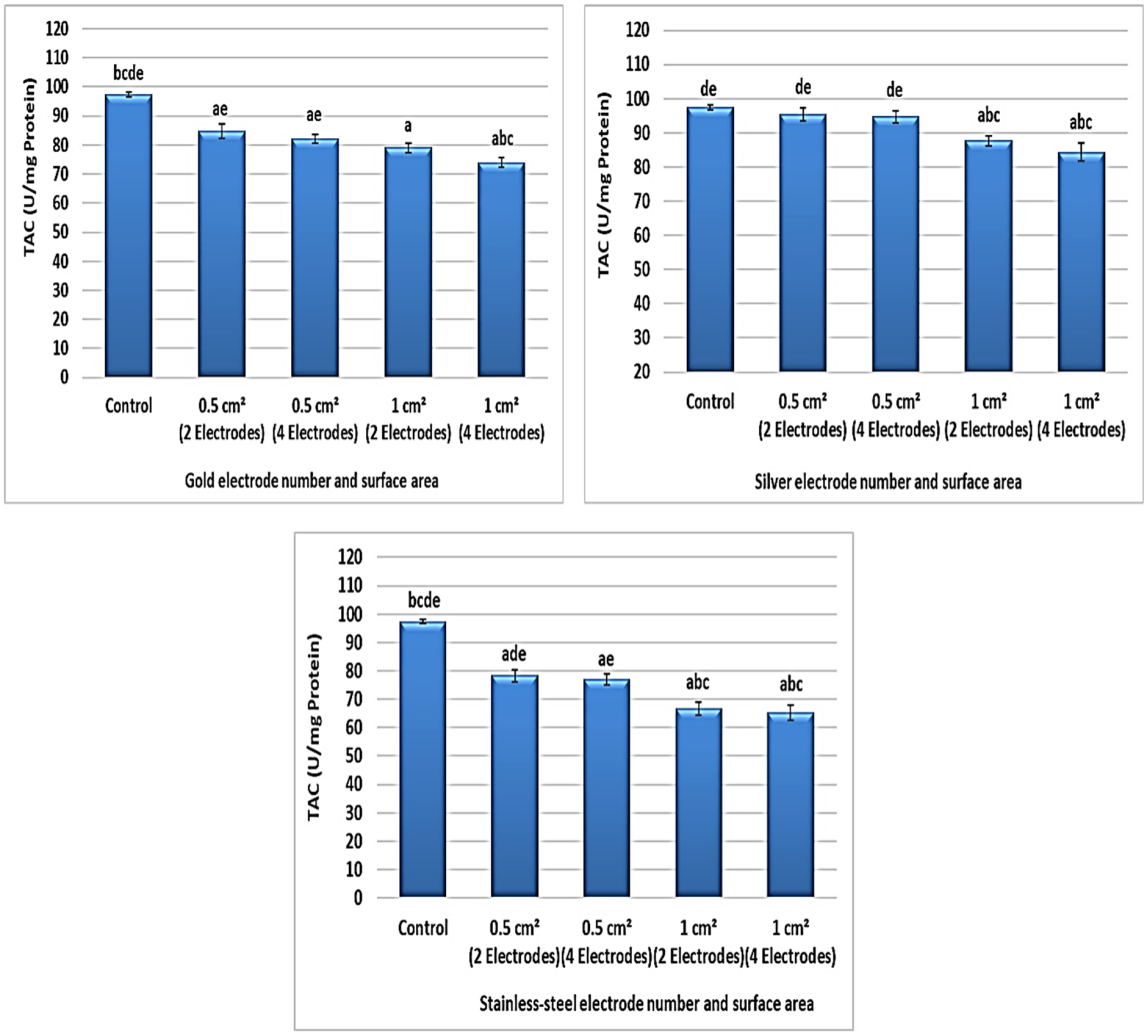
The TAC of MCF-7 cells following TTFields-like exposure conducted by gold electrodes, silver electrodes, and stainless-steel electrodes. Data were expressed as mean ± standard error (SE). Statistical analysis was performed using one-way ANOVA followed by Tukey’s post hoc test. Different superscript letters indicate significant differences at *p* ≤ 0.05. a indicates *p* ≤ 0.05 compared to the ** control** group. b indicates *p* ≤ 0.05 compared to the ** 0.5 cm**^**2**^ **/ 2 electrodes** group. c indicates *p* ≤ 0.05 compared to the ** 0.5 cm**^**2**^ **/ 4 electrodes** group. d indicated *p* ≤ 0.05 compared to the **1 cm**^**2**^ **/ 2 electrodes** group. e indicates *p* < 0.05 compared to the **1 cm**^**2**^ **/ 4 electrodes** group.

One of the primary concerns associated with the use of conductive metal electrodes is the potential induction of oxidative stress resulting from free radical generation. To evaluate electrode-dependent oxidative effects and establish a risk–benefit balance, the TAC of MCF-7 cells was assessed following exposure to TTFields-like compared with control cells (Fig. 5).

Exposure to gold and stainless-steel electrodes resulted in a significant reduction in TAC under all tested electrode configurations (*p* ≤ 0.05). For gold electrodes, TAC decreased from (97.35 ± 0.70 U/mg protein) in control cells to (74.00 ± 1.53 U/mg protein) at 1 cm^2^ with four electrodes. A similar but more pronounced reduction was observed with stainless-steel electrodes, where TAC declined to (65.33 ± 2.60 U/mg protein) under the same conditions.

In contrast, silver electrodes exhibited a substantially lower impact on cellular antioxidant capacity. At a surface area of 0.5 cm^2^, no significant changes in TAC were observed for either two or four electrode configurations compared with control cells. A significant decrease in TAC was detected only when the electrode surface area was increased to 1 cm^2^, reaching (84.33 ± 2.60 U/mg protein) at four electrodes (*p* ≤ 0.05).

Comparison of the maximal oxidative effect across electrode materials revealed that silver electrodes induced the smallest change in TAC, with a Δ reduction of (13.02 U/mg protein), compared with (23.35 U/mg protein) for gold electrodes and (32.02 U/mg protein) for stainless-steel electrodes. These findings indicate that, despite effective anticancer activity, silver electrodes are associated with the lowest degree of oxidative stress, highlighting their favorable safety profile relative to gold and stainless-steel electrodes.

Detailed numerical values and statistical comparisons for TAC measurements are provided in Table 4.

**Table 4:** TAC (U/mg Protein) in MCF-7 cells after exposure to TTFields-like electric fields.

Group	Gold	Silver	Stainless-steel
Control	97.35^bcde^ ± 0.70	97.35^de^ ± 0.70	97.35^bcde^ ± 0.70
0.5 cm^2^ − 2 electrodes	84.67^ae^ ± 2.40	95.33^de^ ± 1.86	78.33^ade^ ± 2.19
0.5 cm^2^ − 4 electrodes	82.0^ae^ ± 1.53	94.67^de^ ± 1.86	77.0^ae^ ± 2.08
1 cm^2^ − 2 electrodes	79.0^a^ ± 1.73	87.67^abc^ ± 1.45	66.67^abc^ ± 2.38
1 cm^2^ − 4 electrodes	74.0^abc^ ± 1.53	84.33^abc^ ± 2.60	65.33^abc^ ± 2.60

Data were expressed as mean ± standard error (SE). Statistical analysis was performed using one-way ANOVA followed by Tukey’s post hoc test. Different superscript letters indicate significant differences at *p* ≤ 0.05.

a indicates *p* ≤ 0.05 compared to the **control** group.

b indicates *p* ≤ 0.05 compared to the **0.5 cm**^**2**^ **/ 2 electrodes** group.

c indicates *p* ≤ 0.05 compared to the **0.5 cm**^**2**^ **/ 4 electrodes** group.

d indicated *p* ≤ 0.05 compared to the **1 cm**^**2**^ **/ 2 electrodes** group.

e indicates *p* < 0.05 compared to the **1 cm**^**2**^ **/ 4 electrodes** group.

### Metal analysis

Metal ion accumulation was quantified using ICP-MS to evaluate the electrochemical contribution of conductive electrodes during TTFields-like exposure. Given the pronounced biological effects observed with silver electrodes compared to gold and stainless-steel electrodes, a comparative elemental analysis was performed across all electrode materials and exposure conditions.

**Fig. 6:**
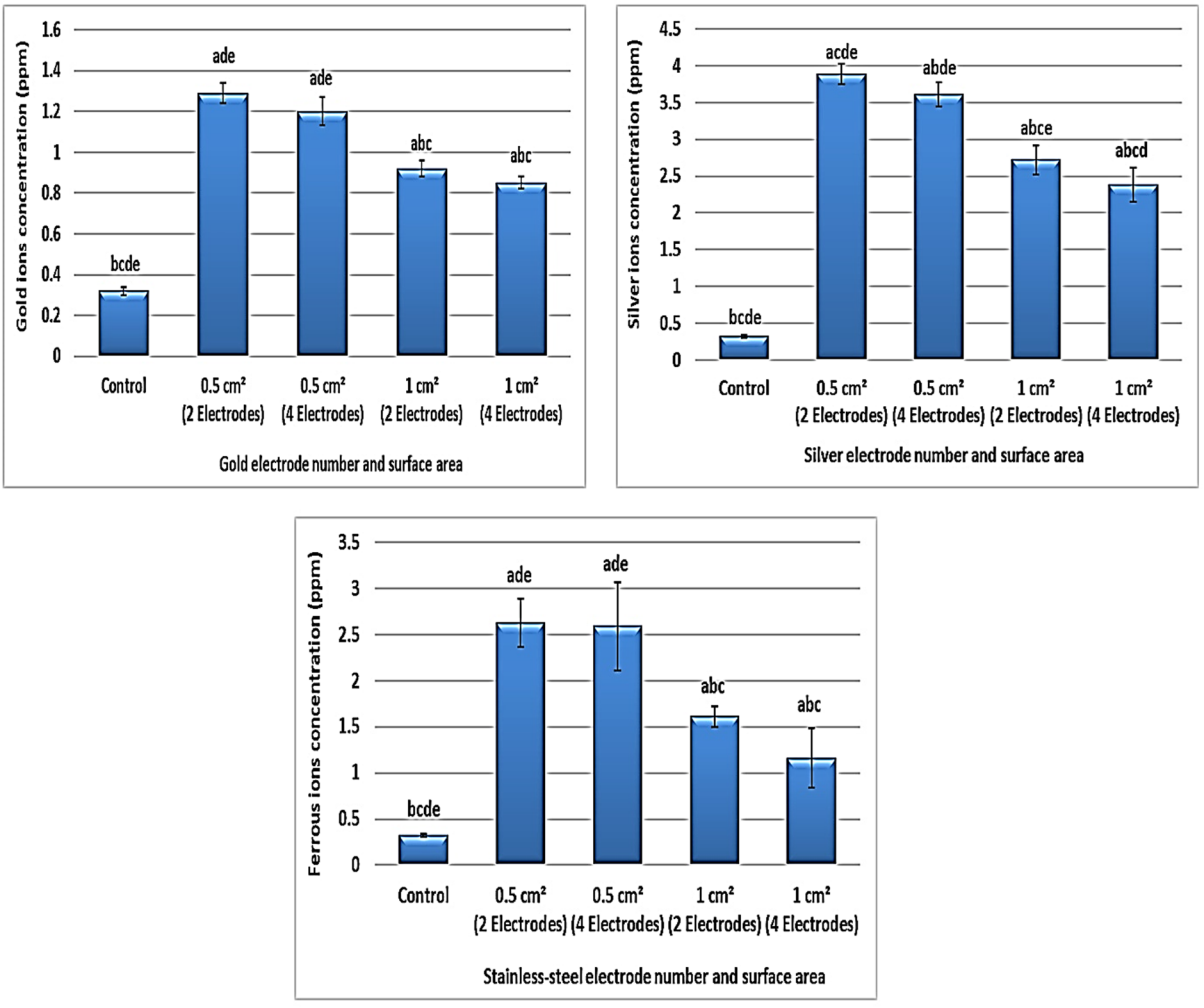
The accumulated ions in the MCF-7 cells following TTFields-like exposure conducted by gold, silver, and stainless-steel electrodes. Data were expressed as mean ± standard error (SE). Statistical analysis was performed using one-way ANOVA followed by Tukey’s post hoc test. Different superscript letters indicate significant differences at *p* ≤ 0.05. a indicates *p* ≤ 0.05 compared to the  **control** group. b indicates *p* ≤ 0.05 compared to the  **0.5 cm**^**2**^ **/ 2 electrodes** group. c indicates *p* ≤ 0.05 compared to the ** 0.5 cm**^**2**^ **/ 4 electrodes** group. d indicated *p* ≤ 0.05 compared to the **1 cm**^**2**^ **/ 2 electrodes** group. e indicates *p* < 0.05 compared to the **1 cm**^**2**^ **/ 4 electrodes** group.

ICP-MS analysis revealed a significant increase in intracellular metal ion concentrations following TTFields-like exposure for all electrode types compared to the control group (Fig. 6 and Supplementary Table S4). However, the magnitude and pattern of ion accumulation differed markedly between materials and exposure configurations.

For gold electrodes, intracellular gold ion levels increased significantly under all exposure conditions, rising from (0.32 ± 0.02) in control cells to (1.29 ± 0.05) at 0.5 cm^2^ / 2 electrodes, and remaining significantly elevated at 0.85 ± 0.03 under 1 cm^2^ / 4 electrodes. Despite this increase, gold ion accumulation demonstrated a progressive decline with increasing electrode surface area and number.

Similarly, silver electrodes exhibited the highest absolute ion accumulation among all materials. Silver ion concentration increased from (0.32 ± 0.02) in controls to (3.89 ± 0.14) at 0.5 cm^2^ / 2 electrodes. Notably, silver ion levels decreased significantly with increasing surface area and electrode number, reaching (2.38 ± 0.23) at 1 cm^2^ / 4 electrodes. Importantly, even under the lowest accumulation condition, silver ion levels remained approximately five-fold higher than control values, indicating sustained ionic release during TTFields-like exposure.

In contrast, stainless-steel electrodes produced moderate ion accumulation, with ferrous ion levels increasing significantly at 0.5 cm^2^ conditions (2.63 ± 0.26 and 2.59 ± 0.48). However, no significant differences were observed between exposure configurations at higher surface areas, and the 1 cm^2^ / 4 electrodes condition showed no significant change compared to the control.

Collectively, these findings demonstrate that while TTFields-like exposure induces metal ion release from all conductive electrodes, silver electrodes combine the highest biological impact with a controlled reduction in ion accumulation at larger surface areas, supporting their favorable risk–benefit profile.

## Discussion

Despite substantial advances in cancer research, achieving durable and selective cancer control remains a major challenge. Multidrug resistance to conventional chemotherapeutic agents continues to limit therapeutic efficacy while increasing systemic toxicity^30–32^. These limitations have driven interest toward non-invasive physical therapies that selectively disrupt cancer cell biology while sparing normal tissues.

Tumor Treating Fields (TTFields) represent a non-invasive anticancer modality that exploits the susceptibility of dividing cancer cells to low-intensity, intermediate-frequency alternating electric fields^33,34^. Although their antimitotic activity is well established, growing evidence indicates that TTFields exert multifaceted intracellular effects involving DNA damage responses, autophagy, membrane permeability, oxidative stress, and apoptosis. Given the abundance of charged and polarizable intracellular components, exposure to TTFields-like electric fields is expected to impose biophysical forces capable of perturbing multiple cellular processes^35–37^.

In the present study, we systematically investigated how electrode material, surface area, and electrode number modulate the biological efficacy of TTFields-like exposure in MCF-7 breast cancer cells. Flow cytometric analysis revealed a consistent and significant reduction in viable (normal) cells across all exposed groups compared with the metal-only control, accompanied by marked increases in apoptotic and necrotic populations. Importantly, these effects were strongly dependent on electrode parameters.

Increasing electrode surface area produced a more pronounced reduction in cell viability than increasing electrode number, irrespective of electrode material. The stronger influence of electrode surface area may be explained by its direct effect on the electrode–electrolyte interface and current distribution. Increasing electrode surface area alters charge density and increases the effective area through which the alternating electric field is delivered to the culture medium. Consequently, modifications in electrode surface area may produce larger changes in cellular exposure conditions than increasing electrode number alone. This observation suggests that electrode geometry represents a critical determinant of biological response in conductive TTFields-like systems. For example, when the electrode number was held constant, doubling the surface area from 0.5 to 1 cm^2^ consistently resulted in a greater decrease in normal cell percentages across gold, silver, and stainless-steel electrodes. This observation aligns with theoretical and experimental studies demonstrating that electrode geometry directly influences electric field distribution, dose density, and charge density at the cellular interface^38^. Larger electrode surface areas promote a more uniform field distribution and enhanced interaction between the electric field and intracellular dipoles, thereby amplifying biological effects.

When comparing electrode materials under identical geometric conditions, silver electrodes consistently produced the strongest anticancer effects, followed by gold and then stainless steel.

The most pronounced reduction in viable cells was observed with four silver electrodes of 1 cm^2^ surface area, resulting in a (~ 20.6%) decrease in normal cells compared with control, exceeding the effects observed with gold and stainless steel under the same conditions. This hierarchy was mirrored by apoptosis measurements, where silver electrodes induced the highest early apoptotic fractions (up to ~ 19.97%), significantly greater than those induced by gold (~ 12.03%) or stainless steel (~ 9.32%) (*p* < 0.001).

The observed ranking of biological activity (silver > gold > stainless steel) likely reflects differences in both the electrical and electrochemical properties of the electrode materials. Silver possesses the highest electrical conductivity among the investigated materials, followed by gold and stainless steel, which may facilitate more efficient coupling of the applied alternating electric field to the cellular microenvironment. Electrode composition can influence charge density, current distribution, electrode polarization, and the interaction of electric fields with intracellular charged and polarizable structures. In addition, the three materials exhibit distinct electrochemical behaviours at the electrode–electrolyte interface. Gold is highly corrosion-resistant and chemically inert, whereas stainless steel forms passive oxide layers that modify charge-transfer processes. Silver exhibits high conductivity together with the potential for limited ion release under electrical stimulation. These material-dependent characteristics may contribute to differences in apoptosis induction, oxidative stress, and intracellular metal accumulation. Consistent with this interpretation, silver electrodes produced the strongest reduction in viable cells, the highest apoptotic fractions, and the greatest increase in the BAX/BCL-2 ratio, whereas gold produced intermediate responses and stainless steel produced the weakest effects. Interestingly, the strongest biological responses were not always associated with the highest intracellular metal ion accumulation, suggesting that ion release alone cannot explain the observed anticancer activity. Rather, the findings support a combined contribution of electric-field-mediated effects and material-dependent electrochemical interactions. Previous modeling and experimental studies have similarly demonstrated that electrode material and geometry influence charge density, field distribution, and biological outcomes^38,39^. These observations identify electrode composition as an important determinant of TTFields-like efficacy and support further optimization of conductive electrode systems for electric-field-based cancer therapy research.

The enhanced performance of silver electrodes may be attributed to their high electrical conductivity, which facilitates more efficient electric field delivery and stronger dielectrophoretic forces during mitosis. Previous modeling and experimental studies have shown that electrode material and shape influence charge density and field homogeneity, ultimately affecting biological outcomes^39,40^. These findings support the rationale for exploring conductive metal electrodes as an alternative to insulated ceramic electrodes traditionally used in clinical TTFields systems, particularly for in vitro optimization studies.

At the molecular level, TTFields-like exposure did not significantly alter the expression of cyclins A, B, or E, indicating that the observed reduction in cell viability was not mediated through cyclin-dependent cell cycle arrest, as reported in some TTFields systems^41,42^. Instead, apoptosis appeared to be the dominant mechanism of cell death. This was supported by a significant downregulation of the anti-apoptotic gene BCL-2, concurrent with upregulation of the pro-apoptotic gene BAX, resulting in a markedly elevated BAX/BCL-2 ratio across exposed groups.

Notably, the increase in the BAX/BCL-2 ratio was most pronounced in silver electrode groups, reaching values exceeding 30-fold over control in the 1 cm^2^ / four-electrode configuration. The BAX/BCL-2 ratio is a well-recognized determinant of mitochondrial outer membrane permeabilization and apoptotic susceptibility^43^, indicating that TTFields-like exposure preferentially activates the intrinsic apoptotic pathway rather than inducing mitotic arrest alone. Although silver electrodes produced the highest BAX/BCL-2 ratio, the magnitude of gene-expression changes did not directly parallel the observed apoptotic-cell percentages. This observation is expected because transcriptional responses represent upstream molecular events, whereas apoptotic-cell accumulation reflects a downstream phenotypic outcome influenced by post-transcriptional regulation, protein synthesis and turnover, signaling-pathway interactions, and temporal differences between gene activation and apoptosis execution. Consequently, changes in the BAX/BCL-2 ratio should not be expected to translate proportionally into apoptotic-cell percentages measured at a single time point. Importantly, both endpoints demonstrated the same overall trend, with silver electrodes producing the strongest pro-apoptotic response, followed by gold and stainless steel.

One concern associated with conductive electrodes is the potential generation of reactive oxygen species (ROS) due to electrochemical reactions at the electrode–electrolyte interface. Assessment of TAC revealed that gold and stainless-steel electrodes induced significant reductions in TAC across all exposure conditions, consistent with increased oxidative stress. In contrast, silver electrodes caused minimal TAC reduction under low-surface-area conditions and exhibited the lowest ΔTAC change even under maximal exposure (1 cm^2^ / four electrodes).

These findings suggest that silver electrodes achieve superior anticancer efficacy while imposing comparatively lower oxidative stress, supporting a favorable risk–benefit balance. This observation aligns with electroporation and pulsed electric field studies demonstrating that electrode material strongly influences ROS generation, with stainless steel producing greater oxidative stress than noble or high-conductivity metals^44^.

ICP-MS analysis confirmed increased intracellular accumulation of gold, silver, and iron ions following TTFields-like exposure. Interestingly, silver ion accumulation decreased with increasing electrode surface area and number, despite enhanced anticancer efficacy. This apparent inverse relationship suggests that biophysical electric field effects, rather than ion toxicity, are the dominant drivers of cell death under the studied conditions. Importantly, unlike direct current (DC) stimulation, the alternating current (AC) fields used in TTFields-like exposure limit net electrochemical reactions at the electrode–electrolyte interface due to charge reversal in each cycle, thereby reducing sustained Faradaic ion release while preserving strong electric field–mediated biological effects^45^. Also, electrode surface area and configuration influence charge density and current distribution, thereby modulating both electric field strength and electrochemical reaction rates^46,47^.

Collectively, these findings demonstrate that optimizing electrode material and geometry significantly enhances the biological efficacy of TTFields-like electric fields. Silver electrodes with larger surface areas provide the strongest anticancer effects through apoptosis induction, while minimizing oxidative stress and limiting excessive ion accumulation. However, the maximal reduction in viable cells (~ 73.6%) indicates that further optimization is possible. Because conductive metallic electrodes were employed, the observed biological responses likely reflect contributions from both electric-field-mediated mechanisms and electrode-associated electrochemical processes. However, several observations indicate that electrochemical toxicity alone is insufficient to explain the results. The magnitude of intracellular metal accumulation did not consistently parallel the observed reductions in cell viability or increases in apoptosis across all electrode configurations. In addition, biological efficacy was strongly influenced by electrode geometry, particularly electrode surface area, suggesting an important contribution of electric-field delivery conditions. These findings support the interpretation that the observed anticancer responses arise from the combined influence of electric-field-mediated cellular effects and material-dependent electrochemical interactions rather than from metal-ion toxicity alone.

A key limitation of the present study is the absence of frequency optimization, a parameter known to be highly cell-line dependent in TTFields therapy. Future studies integrating electrode optimization with frequency tuning, computational electric field modeling, and in vivo validation may further improve therapeutic outcomes and advance the rational design of electric field–based anticancer strategies. Another limitation is that only one breast cancer cell line (MCF-7) was evaluated, as breast cancer is biologically heterogeneous, future studies should validate these findings in additional cell lines representing different molecular subtypes. Also, the present study is that only conductive metallic electrode configurations were investigated. Consequently, the observed biological responses reflect the behavior of a conductive electrode-based TTFields-like exposure platform and cannot be assumed to directly represent the response that would occur in insulated transducer-array TTFields systems. Future studies comparing conductive and insulated electrode configurations under matched exposure conditions would be valuable for distinguishing electric-field-mediated effects from electrode-associated electrochemical contributions and for determining the extent to which the present findings translate to conventional TTFields applications. Furthermore, apoptosis-related markers were evaluated at the mRNA level only. Protein-level validation of BAX, BCL-2, and related apoptotic proteins was beyond the scope of the present study. Consequently, the observed transcriptional changes should be interpreted as evidence of modulation of apoptosis-related pathways rather than direct quantification of protein abundance or activity.

## Conclusion

TTFields-like exposure generated using conductive metallic electrodes represents a promising in vitro electric-field-based anticancer approach whose biological activity is influenced by electrode-related parameters. The present study demonstrates that electrode material, number, and surface area significantly affect cellular responses in MCF-7 breast cancer cells under the investigated exposure conditions.

Among the tested materials, silver electrodes produced the strongest anticancer responses, characterized by reduced cell viability, increased apoptosis, favorable modulation of BAX and BCL-2 gene expression, and distinct patterns of oxidative stress and intracellular metal accumulation. Increasing electrode number and electrode surface area generally enhanced these biological effects.

Collectively, these findings demonstrate that electrode material and geometry are important determinants of biological response in conductive electrode-based TTFields-like systems. The results provide a foundation for further optimization and characterization of such exposure platforms and support future studies aimed at distinguishing electric-field-mediated effects from electrode-associated electrochemical interactions.

## Supplementary Information

Below is the link to the electronic supplementary material.

Supplementary Material 1

Supplementary Material 2

Supplementary Material 3

Supplementary Material 4

## Data Availability

All data generated or analyzed during this study are included in this published article and the attached Supplementary Materials.
