# Supplementary material for: Optimization of electrode material and geometry to enhance TTFields-like anticancer effects in MCF-7 breast cancer cells
Source: Sci Rep. 2026 Aug 13;16:25290. doi: 10.1038/s41598-026-65772-2 (PMC13473482; doi:10.1038/s41598-026-65772-2)
Supplement: Supplementary file 1 [file 41598_2026_65772_MOESM1_ESM.docx]

**Table S1. The relative amount of BAX, BCL-2, and the BAX/BCL-2 ratio in MCF-7 cells after exposure to TTFields-like electric fields conducted through gold electrodes compared to the control.**

| **Group** | **BAX** | **BCL-2** | **BAX/BCL-2** |
| --- | --- | --- | --- |
| **Control** | 1.00^bcde^ ± 0.02 | 1.00^bcde^ ± 0.02 | 1.00 ^bcde^ ± 0.02 |
| **0.5 cm^2^ - 2 electrodes** | 1.74^ade^ ± 0.10 | 0.85^ade^ ± 0.05 | 2.05^ade^ ± 0.08 |
| **0.5 cm^2^ - 4 electrodes** | 2.03^ade^ ± 0.05 | 0.75^ade^ ± 0.08 | 2.72^ade^± 0.23 |
| **1 cm^2^ - 2 electrodes** | 4.10^abc^ ± 0.27 | 0.45^abc^ ± 0.07 | 9.2^abc^ ± 0.84 |
| **1 cm^2^ - 4 electrodes** | 4.31^abc^ ± 0.13 | 0.40^abc^ ± 0.01 | 10.86^abc^ ± 0.10 |

The significance between groups is determined by the Kruskal-Wallis test; data were expressed in mean ± standard error.

a indicates p≤0.05 compared to the **control** group.

b indicates p ≤ 0.05 compared to the **0.5 cm^2^ / 2 electrodes** group.

c indicates p ≤ 0.05 compared to the **0.5 cm^2^ / 4 electrodes** group.

d indicated p≤ 0.05 compared to the **1 cm^2^ / 2 electrodes** group.

e indicates p < 0.05 compared to the **1 cm^2^ / 4 electrodes** group.
