# Supplementary material for: Optimization of electrode material and geometry to enhance TTFields-like anticancer effects in MCF-7 breast cancer cells
Source: Sci Rep. 2026 Aug 13;16:25290. doi: 10.1038/s41598-026-65772-2 (PMC13473482; doi:10.1038/s41598-026-65772-2)
Supplement: Supplementary file 2 [file 41598_2026_65772_MOESM2_ESM.docx]

**Table S2. The relative amount of BAX, BCL-2, and the BAX/BCL-2 ratio in MCF-7 cells after exposure to TTFields-like electric fields conducted through silver electrodes compared to the control.**

| **Group** | **BAX** | **BCL-2** | **BAX/BCL-2** |
| --- | --- | --- | --- |
| **Control** | 1.00^bcde^ ±0.02 | 1.00^bcde^ ± 0.02 | 1.00^bcde^ ± 0.02 |
| **0.5 cm^2^ - 2 electrodes** | 1.98^ade^ ±0.10 | 0.7^ade^ ± 0.09 | 2.85^ade^ ± 0.56 |
| **0.5 cm^2^ - 4 electrodes** | 2.90^ade^ ± 0.35 | 0.65^ade^ ± 0.12 | 4.5^ade^ ± 0.30 |
| **1 cm^2^ - 2 electrodes** | 6.47^abc^ ± 0.35 | 0.29^abc^ ± 0.04 | 22.49^abc^ ± 1.91 |
| **1 cm^2^ - 4 electrodes** | 7.52^abc^ ± 0.26 | 0.24^abc^ ± 0.06 | 30.68^abc^ ± 4.46 |

The significance between groups is determined by the Kruskal-Wallis test; data were expressed in mean ± standard error.

a indicates p≤0.05 compared to the **control** group.

b indicates p ≤ 0.05 compared to the **0.5 cm^2^ / 2 electrodes** group.

c indicates p ≤ 0.05 compared to the **0.5 cm^2^ / 4 electrodes** group.

d indicated p≤ 0.05 compared to the **1 cm^2^ / 2 electrodes** group.

e indicates p < 0.05 compared to the **1 cm^2^ / 4 electrodes** group.
