# Supplementary material for: Optimization of electrode material and geometry to enhance TTFields-like anticancer effects in MCF-7 breast cancer cells
Source: Sci Rep. 2026 Aug 13;16:25290. doi: 10.1038/s41598-026-65772-2 (PMC13473482; doi:10.1038/s41598-026-65772-2)
Supplement: Supplementary file 4 [file 41598_2026_65772_MOESM4_ESM.docx]

**Table S4. The concentration of silver ions (ppm) in MCF-7 cells after exposure to TTFields-like electric fields.**

| **Group** | **Gold** | **Silver** | **Stainless-steel** |
| --- | --- | --- | --- |
| **Control** | 0.32^bcde^ ± 0.02 | 0.32^bcde^ ± 0.02 | 0.32^bcde^ ± 0.02 |
| **0.5 cm^2^ - 2 electrodes** | 1.29^ade^ ± 0.05 | 3.89^ade^ ± 0.14 | 2.63^ade^ ± 0.26 |
| **0.5 cm^2^ - 4 electrodes** | 1.20^ade^ ± 0.07 | 3.61^ade^ ± 0.16 | 2.59^ade^ ± 0.48 |
| **1 cm^2^ - 2 electrodes** | 0.92^abc^ ± 0.04 | 2.72^abc^ ± 0.20 | 1.61^abc^ ± 0.11 |
| **1 cm^2^ - 4 electrodes** | 0.85^abc^ ± 0.03 | 2.38^abc^ ± 0.23 | 1.16^abc^ ± 0.32 |

Data were expressed as mean ± standard error (SE). Statistical analysis was performed using one-way ANOVA followed by Tukey’s post hoc test. Different superscript letters indicate significant differences at p ≤ 0.05.

a indicates p≤0.05 compared to the **control** group.

b indicates p ≤ 0.05 compared to the **0.5 cm^2^ / 2 electrodes** group.

c indicates p ≤ 0.05 compared to the **0.5 cm^2^ / 4 electrodes** group.

d indicated p≤ 0.05 compared to the **1 cm^2^ / 2 electrodes** group.

e indicates p < 0.05 compared to the **1 cm^2^ / 4 electrodes** group.
